# Alginate oligosaccharide extends the service lifespan by improving the sperm metabolome and gut microbiota in an aging Duroc boars model

**DOI:** 10.3389/fcimb.2023.1308484

**Published:** 2023-12-05

**Authors:** Yexun Zhou, Zeou Wei, Jiajian Tan, Haiqing Sun, Haidi Jiang, Yang Gao, Hongfu Zhang, Martine Schroyen

**Affiliations:** ^1^ State Key Laboratory of Animal Nutrition, Institute of Animal Sciences, Chinese Academy of Agricultural Sciences, Beijing, China; ^2^ Precision Livestock and Nutrition Unit, Gembloux Agro-Bio Tech, University of Liège, Gembloux, Belgium; ^3^ YangXiang Joint Stock Company, Animal Nutrition Institute, Guigang, China; ^4^ College of Life Science, Baicheng Normal University, Baicheng, Jilin, China

**Keywords:** alginate oligosaccharide, service lifespan, gut microbiota, sperm metabolome, aging Duroc boars

## Abstract

**Introduction:**

Alginate oligosaccharide (AOS), as a natural non-toxic plant extract, has been paid more attention in recent years due to its strong antioxidant, anti-inflammatory, and even anti-cancer properties. However, the mechanism by which AOS affects animal reproductive performance is still unclear.

**Methods:**

The purpose of this study is to use multi-omics technology to analyze the effects of AOS in extending the service lifespan of aging boars.

**Results:**

The results showed that AOS can significantly improve the sperm motility (*p* < 0.05) and sperm validity rate (*p* < 0.001) of aging boars and significantly reduce the abnormal sperm rate (*p* < 0.01) by increasing the protein levels such as CatSper 8 and protein kinase A (PKA) for semen quality. At the same time, AOS significantly improved the testosterone content in the blood of boars (*p* < 0.01). AOS significantly improved fatty acids such as adrenic acid (*p* < 0.05) and antioxidants such as succinic acid (*p* < 0.05) in sperm metabolites, significantly reducing harmful substances such as dibutyl phthalate (*p* < 0.05), which has a negative effect on spermatogenesis. AOS can improve the composition of intestinal microbes, mainly increasing beneficial bacteria *Enterobacter* (*p =* 0.1262) and reducing harmful bacteria such as *Streptococcus* (*p* < 0.05), *Prevotellaceae_UCG-001* (*p* < 0.05), and *Prevotellaceae_NK3B31_group* (*p* < 0.05). Meanwhile, short-chain fatty acids in feces such as acetic acid (*p* < 0.05) and butyric acid (*p* < 0.05) were significantly increased. Spearman correlation analysis showed that there was a close correlation among microorganisms, sperm metabolites, and sperm parameters.

**Discussion:**

Therefore, the data indicated that AOS improved the semen quality of older boars by improving the intestinal microbiota and sperm metabolome. AOS can be used as a feed additive to solve the problem of high elimination rate in large-scale boar studs.

## Introduction

In recent years, the research on breeding pigs is no longer limited to nutritional demands; more importantly, the problems of reproductive performance and service lifespan have already been focused on more widely (Poulsen et al., 2020; Plaengkaeo et al., 2021). Excellent breeding boars can directly affect the benefits of pig farms. Therefore, a reasonable extending service lifespan has important practical significance in terms of production in the swine industry (Spinaci et al., 2016), such as saving breeding costs, increasing conception rate and litter sizes, and improving the stability of the whole swine population (D’Allaire et al., 1992; Hoffman and Valencak, 2020). The service lifespan of breeding boars refers to from the first mating to elimination (Koketsu and Sasaki, 2009). A study has shown that the average service lifespan of boars in the late 20th century was 20 months (D’Allaire and Leman, 1990). In the early 21st century, it was mainly concentrated in 2 years (Knox et al., 2008). In China, the lifespan of breeding boars in large-scale boar studs is currently 30 months. However, there are many factors affecting the service lifespan, such as varieties, nutrition, and environment (Cassady et al., 2002; Sancho et al., 2004; Akerfelt et al., 2010). These factors resulted in worse semen quality, decreased sexual desire (Berger et al., 1980), and eventually death. Therefore, we hope to improve the breeding performance of boars through nutritional regulation and determine the appropriate feed formula so as to extend its service lifespan.

Alginate oligosaccharide (AOS) is a natural and non-toxic plant extract that comes from alginate (Li et al., 2022). Because of its multiple biological properties, such as anti-inflammatory (Feng et al., 2020), anti-cancer (Han et al., 2019), and antioxidant (Zhang et al., 2022b), it is currently widely used in the medical field. AOS can activate the specific immune system and inhibit the proliferation of tumor cells by activating macrophages (Saigusa et al., 2015). AOS can not only remove active oxygen, but also significantly reduce the content of lipid peroxidation. At the same time, it can increase the activity of hydrogen peroxide and superoxide dismutase (SOD), thus removing excessive free radicals (Zhao et al., 2020a). Studies have shown that AOS can alleviate the intestinal inflammation of DSS-induced mice, which is conducive to improving the intestinal health of animals (Zhang et al., 2022a; Lu et al., 2023). Our previous study found that AOS can repair the testicular damage of mice induced by Busulfan, thereby improving the semen quality (Zhang et al., 2021a). However, few research reported that AOS can extend the lifespan of aging animals. The purpose of the study is to explore the potential mechanism of AOS to extend the lifespan of aging boars, then provide a theoretical basis for solving the problem of high elimination rates in large-scale boar studs.

## Materials and methods

### Boars and experimental design

**Figure 1:**
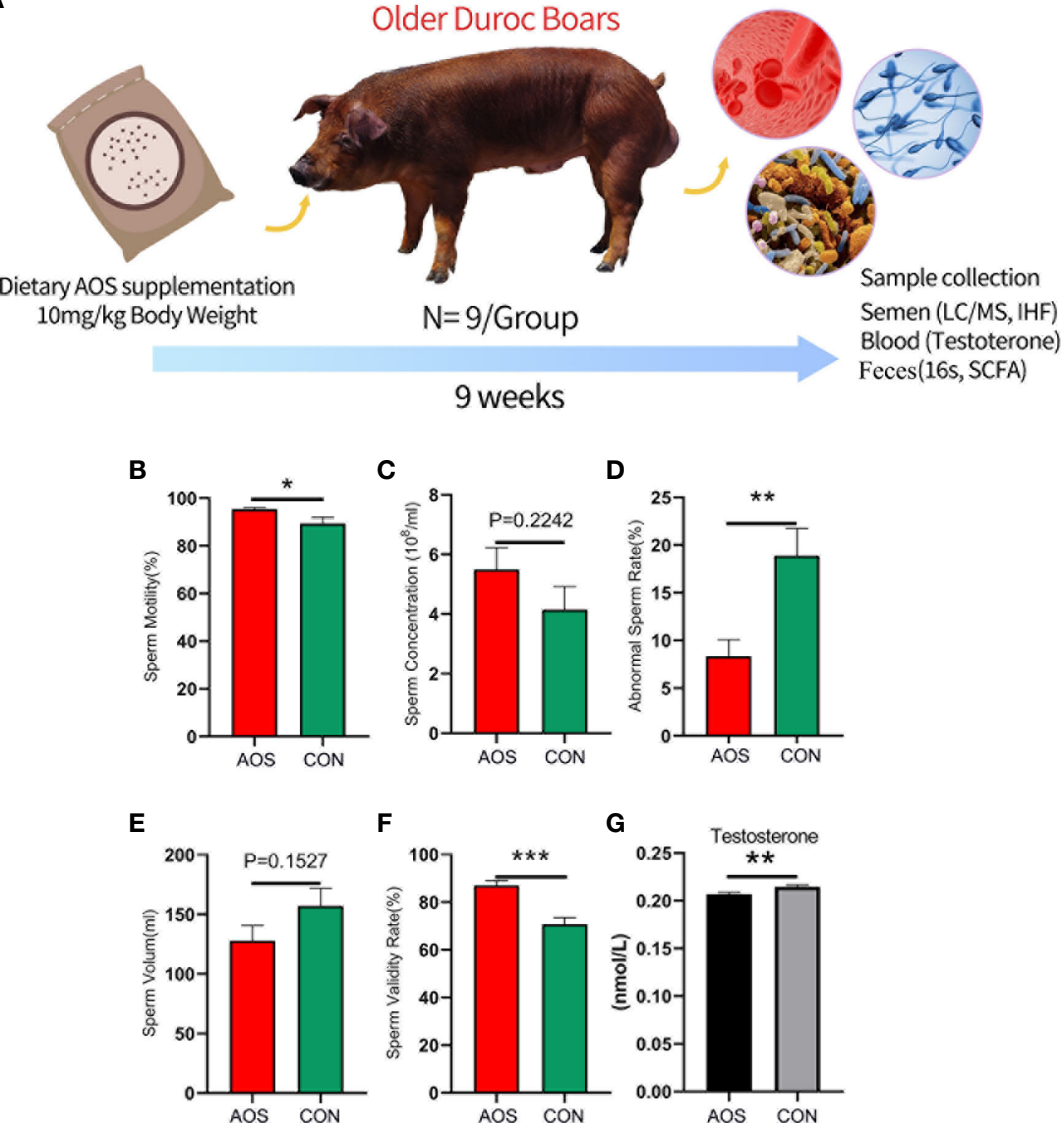
AOS improved the semen parameters and blood testosterone content of aging boars. **(A)** Study design. **(B)** Sperm motility. The *y*-axis represents the percentage of total cells. The *x*-axis represents the treatment (*n* = 9/group). ^*^ *p* < 0.05. **(C)** Sperm concentration. The *y*-axis represents the concentration. The *x*-axis represents the treatment (*n* = 9/group). *p =* 0.2242. **(D)** Abnormal sperm rate. The *y*-axis represents the percentage of abnormal cells. The *x*-axis represents the treatment (*n* = 9/group). ^**^ *p* < 0.01. **(E)** Sperm volume. The *y*-axis represents the volumetric weight. The *x*-axis represents the treatment (*n* = 9/group). *p =* 0.1527. **(F)** Sperm validity rate. The *y*-axis represents the percentage of validity cells. The *x*-axis represents the treatment (*n* = 9/group).^***^ *p* < 0.001. **(G)** Blood testosterone content. The *y*-axis represents the testosterone level. The *x*-axis represents the treatment (*n* = 9/group). ^**^ *p* < 0.01. Data were expressed as the mean ± SEM.

The animal experiments followed the guidelines from the Animal Care and Use Committee of the Institute of Animal Sciences, Chinese Academy of Agricultural Sciences (IAS2022-24). Eighteen Duroc boars of similar age (65 months old), health status, and body weight (approximately 300 kg) were selected randomly from the Ya Ji Mountain boar stud facility for the investigation. The Duroc boars were randomly divided into two groups, namely, the control (CON) group and the AOS group, with each group consisting of nine boars. The CON group was fed a basal diet (feed formula is shown in **Table S1**), and boars in the AOS group were fed a basal diet with 10 mg of AOS per kg of body weight (Han et al., 2022) (provided by Qingdao Zhibo Biotechnology Co., Ltd). Each boar was housed in an individual pen and the whole feeding period lasted 9 weeks (**Figure 1A**).

In the experiment, we used gloved-hand technology to obtain the semen samples. After that, sperm parameters including sperm concentration, sperm motility, abnormal sperm rate, sperm volume, and sperm validity rate were assessed by CASAII software according to the reported methods (Guo et al., 2020). Blood samples were taken from hind leg veins when the animals were ejaculating and placed in an anti-coagulated tube. Then, blood samples were centrifuged at 3,000 × *g* for 10 min, the supernatant was transferred to a 1.5-mL centrifuge tube, and the plasma was stored in a −80°C refrigerator for further research. Fecal samples were taken from the rectum by hand, the rectum of the boars was massaged to promote peristalsis and to obtain fresh feces, which were placed in liquid nitrogen immediately and finally stored in a −80°C freezer for 16S sequencing analysis and short-chain fatty acid test.

### Using a computer-assisted sperm analysis system to detect sperm parameters

The sperm parameters, including sperm concentration, sperm motility, abnormal sperm rate, sperm volume, and sperm validity rate, were analyzed by a computer-assisted sperm analysis (CASAII) system (Shanghai Kasu Biotechnology Co., Ltd., Shanghai, China). Evaluation criteria for sperm motility were as follows: grade A, fast forward movement > 22 μm s^−1^; grade B, forward movement < 22 μm s^−1^; grade C, curve movement < 5 μm s^−1^; grade D, no movement (Yeste et al., 2018). The sperm concentration should be more than 10^8^/mL and the abnormal sperm rate should be less than 30%. The semen volume should not be less than 50 mL each time. The sperm validity rate should be more than 80% (Cao et al., 2011).

### Detection of blood testosterone content

Blood testosterone content was measured by ELISA kits (Beijing Boxbio Science & Technology Co., Ltd) following the instructions of the manufacturer. Then, the microplate reader (central laboratory) was used to detect the absorbance value of each sample. Finally, the plasma testosterone content was calculated by using the formula based on the instructions.

### Sperm metabolome assay by LC-MS/MS

Boar sperm (*n* = 6 per group) was taken out from a −80°C fridge. Firstly, the protein was removed from the samples and then analyzed by LC/MS. Next, An ACQUITY UPLC BEH C18 column (1.7 μm, 2.1 × 100 mm) was employed in both positive and negative modes. Solvent A is an aqueous solution containing 0.1% formic acid. Solvent B is an aqueous solution containing 0.1% acetonitrile. The following program was followed: 5%–20% B over 0–2 min; 20%–60% B over 2–4 min; 60%–100% B over 4–11 min; the composition was held at 100% B for 2 min, then 13–13.5 min, 100% to 5% B, and 13.5–14.5 min holding at 5% B. The flow rate was set at 0.4 mL/min and the column temperature was 45°C. The sperm was kept at 4°C and the volume of the injection was 5 μL. ESI was used in the mass spectrometry program.

### Using immunofluorescence staining to detect the protein levels in boar sperm

The IHF methods for boar sperm have been reported in our previous articles (*n* = 9 per group) (Zhou et al., 2022). Primarily, the boar sperm was fixed in 4% paraformaldehyde for 1 h, then air-dried and spread on the slides covered with poly-L-lysine. After washing three times (5 min each) with PBS, the sperm was incubated with 2% Triton X-100 in PBS for 1 h at room temperature. Next, the sperm was washed three times (5 min each) again with PBS, and was blocked with PBS, which contained 1% BSA and 1% goat serum, for 30 min at 17°C. This was followed by incubation with diluted primary antibody (1:100; **Table S2**) overnight at 4°C. The next morning, the sperm was washed three times with PBS that contained 1% BSA (the secondary antibody dilution), each time for 5 min. Secondary antibody (1:100) was added to the diluent and incubated at 37°C in the dark for 1 h. This was followed by washing three times with PBS (5 min each); Hoechst 33342 was added to stain the nucleus, with a waiting time of 5 min at room temperature. Then, the sperm was again washed three times with PBS for 5 min each time, the accelerator was added, and pictures were taken using a fluorescence microscope (LEICA TCS SP5 II, Germany). The protein positive rate = red sperm/total sperm × 100% in the view, which was selected randomly. Each slide was chosen 5 different screens, then made a calculation to gain the positive rate.

### Boar feces 16s RNA sequencing and short-chain fatty acid test

The protocol for the analysis of fecal microbiota was reported in our previous study (Zhou et al., 2022) (*n* = 9 per group).

An E.Z.N.A. ^®^ Stool DNA Kit (Omega Bio-tek Inc., USA) was used to separate total fecal genomic DNA, following the manufacturer’s instructions. NanoDrop 2000 (Thermo Scientific, USA) and 1% agarose gel were used to detect the DNA quantity and quality, respectively. Primer pairs 338F (5’-ACTCCTACGGGAGGCAGCAG-3’) and 806R (5’-GGACTACHVGGGTWTCTAAT-3’) were used to amplify the V3–V4 region of the microbial 16S rRNA genes. PCR system and amplification conditions followed those from our previous study (Wan et al., 2021). The PCR amplification products can be extracted by 2% agarose gel, and the AxyPrep DNA Gel Extraction Kit (AXYGEN, New York, NY, United States) was used to purify them following the manufacturer’s instructions. After that, the sequences were assigned to the same operational taxonomic units (OTUs > 97% similarity).

Concentrations of SCFAs in feces were measured by using GC-MS. Briefly, fecal samples were placed into 1.5-mL centrifuge tubes and mixed with 1 mL of ddH_2_O, homogenized, and centrifuged (10,000 rpm, 10 min, 4°C). A mixture of the supernatant fluid and 25% metaphosphoric acid solution (0.9 and 0.1 mL, respectively) was vortexed for 1 min and centrifuged (1,000 rpm, 10 min, 4°C) after being placed in a 1.5-mL centrifuge tube at 4°C for over 2 h. The supernatant portion was then filtered through a 0.45-μm polysulfone filter and analyzed using Agilent 6890 gas chromatography (Agilent Technologies, Inc., Palo Alto, CA, United States).

### Statistical analysis

Data are expressed as the mean ± SEM. *p* < 0.05 was considered statistically significant. The Student’s *t*-test (SPSS 21 software) was used to perform the statistical analyses. Spearman’s correlation analysis was completed by the RStudio (version 4.0.3) platform. Plots were performed using GraphPad Prism 8.0.2.

## Results

### AOS improved semen parameters and blood testosterone content of aging boars

As shown in **Figure 1A** (Study scheme), the aging Duroc boars were fed AOS (10 mg/kg body weight) for 63 days. Dietary supplementation of AOS significantly increased the sperm motility (**Figure 1B**; *p* < 0.05). Meanwhile, the abnormal sperm rate was significantly decreased compared to the CON group (**Figure 1D**; *p* < 0.01). In contrast, the sperm validity rate of the AOS group was significantly higher than that of the CON group (**Figure 1F**; *p* < 0.001). Apart from that, the sperm concentration (**Figure 1C**; *p =* 0.2242) and sperm volume (**Figure 1E**; *p =* 0.1527) were not different between the two groups. Adding AOS in the basal diet can significantly increase the testosterone content in the blood of the aging boar (**Figure 1G**; *p* < 0.01).

### AOS improved the protein associated with the spermatogenesis of aging boars

**Figure 2:**
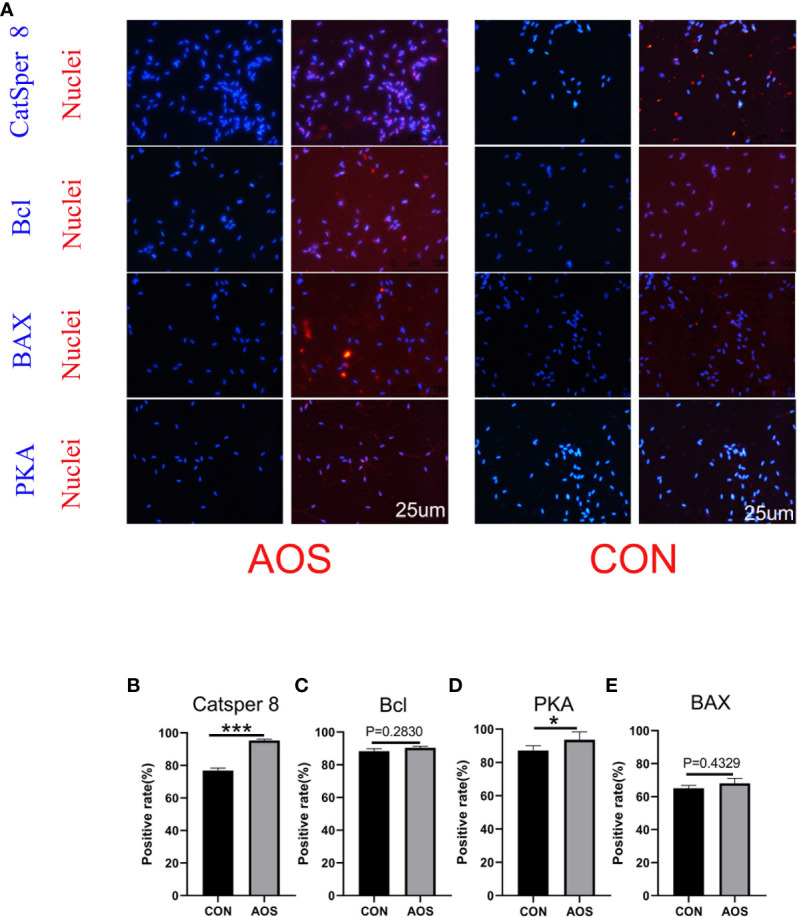
AOS improved the protein related to spermatogenesis of aging boars. **(A)** Immunofluorescence staining (IHF) of Catsper 8, Bcl, BAX, and PKA. **(B)** Positive rate of Catsper 8. **(C)** Positive rate of Bcl. **(D)** Positive rate of BAX. **(E)** Positive rate of PKA. Data were expressed as the mean ± SEM. The *y*-axis represents the amount of positive rate. The *x*-axis represents the treatments. (*n* = 9/group) ^***^ *p* < 0.001, ^**^ *p* < 0.01.

In order to understand how AOS prolongs the lifespan of aging Duroc boars, the protein levels (CatSper 8, PKA, Bcl, and Bcl-2-associated X protein) were quantified for sperm quality and spermatogenesis (**Figure 2A**). AOS increased the protein level, which reflected the significantly positive rate of CatSper 8 (**Figure 2B**; *p* < 0.001) and PKA (**Figure 2D**; *p* < 0.01) compared to the CON group by IHF staining. At the same time, AOS improved Bcl (**Figure 2C**; *p =* 0.2830) and Bax (**Figure 2E**; *p =* 0.4329) protein levels, but the differences were not significant.

### AOS improved the sperm metabolites of aging boars

**Figure 3:**
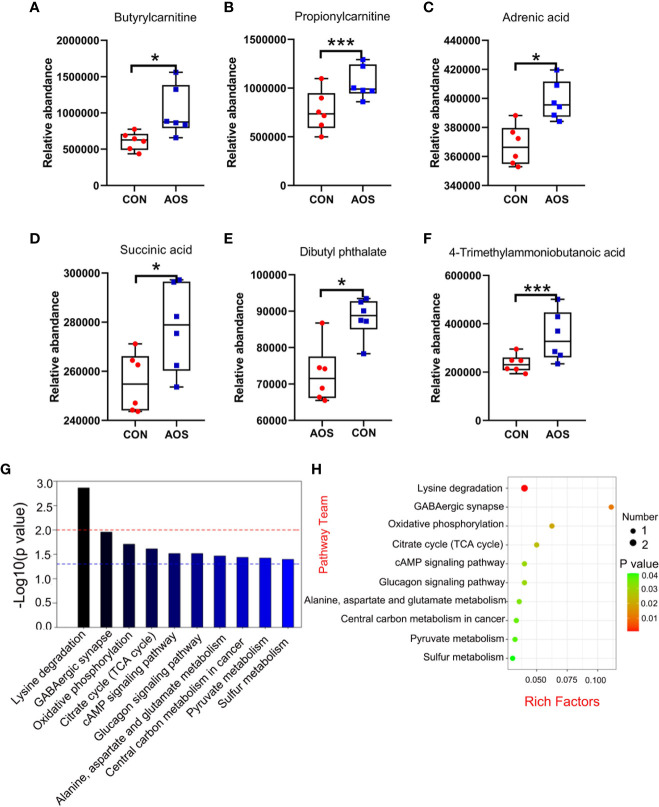
AOS improved the sperm metabolites of aging boars. **(A)** Sperm butyrylcarnitine level. **(B)** Sperm propionylcarnitine level. **(C)** Sperm adrenic acid level. **(D)** Sperm succinic acid level. **(E)** Sperm dibutyl phthalate level. **(F)** Sperm 4-trimethylammoniobutanoic acid level. **(G)** KEGG metabolic pathway histogram (top 10); the red dotted line means *p* < 0.01, and the blue dotted line means *p* < 0.05. **(H)** KEGG metabolic pathway bubble chart (top 10). Data were expressed as the mean ± SEM. The *y*-axis represents the relative amount. The *x*-axis represents the treatments (*n* = 6/group). ^***^ *p* < 0.001, ^*^ *p* < 0.05.

AOS benefited the sperm metabolites, as determined by LC/MS analysis (**Table S3**). Firstly, AOS can significantly increase some types of fatty acids and sperm derivatives such as butyrylcarnitine (**Figure 3A**; *p* < 0.05), propionylcarnitine (**Figure 3B**; *p* < 0.001), adrenic acid (**Figure 3C**; *p* < 0.05), and 4-trimethylammoniobutanoic acid (**Figure 3F**; *p* < 0.001). Secondly, AOS elevated a batch of sperm antioxidants such as succinic acid (**Figure 3D**; *p* < 0.05). Thirdly, AOS can significantly reduce harmful metabolites related to the reproductive function in sperm of aging boars such as dibutyl phthalate (**Figure 3E**; *p* < 0.05). Meanwhile, the potential metabolic pathways of the changed metabolites were determined by KEGG pathway analysis. The top 10 pathways (**Figures 3G, H**; **Table S4**) showed that the changed metabolites were involved in lysine degradation (*p* < 0.01), GABAergic synapse (*p* < 0.05), oxidative phosphorylation (*p* < 0.05), citrate cycle (*p* < 0.05), cAMP signaling pathway (*p* < 0.05), glucagon signaling pathway (*p* < 0.05), alanine, aspartate, and glutamate metabolism (*p* < 0.05), central carbon metabolism in cancer (*p* < 0.05), pyruvate metabolism (*p* < 0.05), and sulfur metabolism (*p* < 0.05).

### AOS changed microbial composition of feces of aging boars

**Figure 4:**
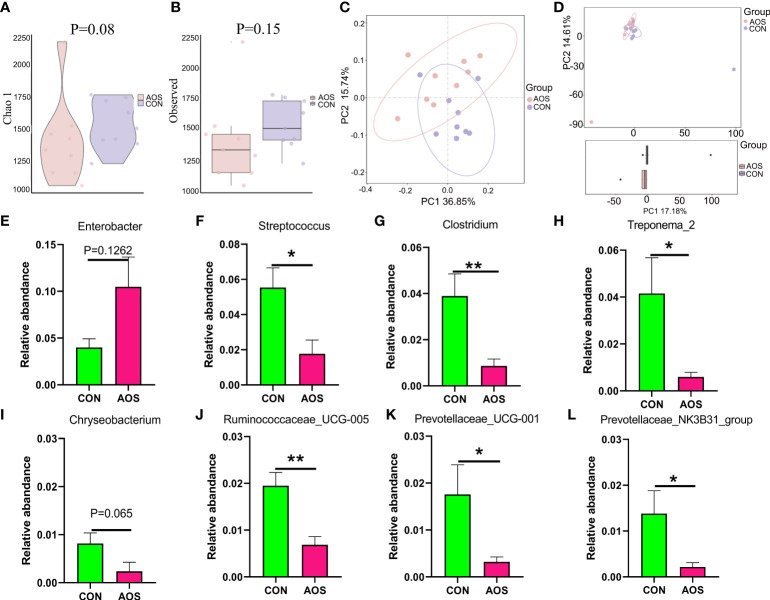
AOS changed microbial composition in the feces of aging boars. **(A)** α-diversity with the Chao 1 level. **(B)** α-diversity with the observed level. **(C)** β-diversity with the PCoA level. **(D)** β-diversity with the PCA level. The relative amount of individual microbiota in feces at the genus level **(E–L)**. Data were expressed as the mean ± SEM. The *y*-axis represents the relative amount. The *x*-axis represents the treatments (*n* = 9/group). ^**^ *p* < 0.01, ^*^ *p* < 0.05.

To investigate the effect of AOS on intestinal microbes of aging boars, we conducted 16S sequencing on feces. The microbes (β-diversity) were different between the AOS and CON groups based on PCA (**Figures 4C, D**). The α-diversity on the Chao1 (**Figure 4A**; *p =* 0.08) and observed (**Figure 4B**; *p =* 0.15) level did not change much; however, there is a trend of changing. AOS increased the abundance of beneficial microbiota at the genus level (**Table S5**) such as *Enterobacter* (**Figure 4E**; *p =* 0.1262). At the same time, AOS decreased the abundance of harmful microbiota such as *Streptococcus* (**Figure 4F**; *p* < 0.05), *Clostridium* (**Figure 4G**; *p* < 0.01), *Treponema_2* (**Figure 4H**; *p* < 0.05), *Chryseobacterium* (**Figure 4I**; *p =* 0.065), *Ruminococcaceae_UCG-005* (**Figure 4J**;*p* < 0.01), *Prevotellaceae_UCG-001* (**Figure 4K**; *p* < 0.05), and *Prevotellaceae_NK3B31_group* (**Figure 4L**; *p* < 0.05).

### AOS improved the content of short-chain fatty acids in aging boar feces

**Figure 5:**
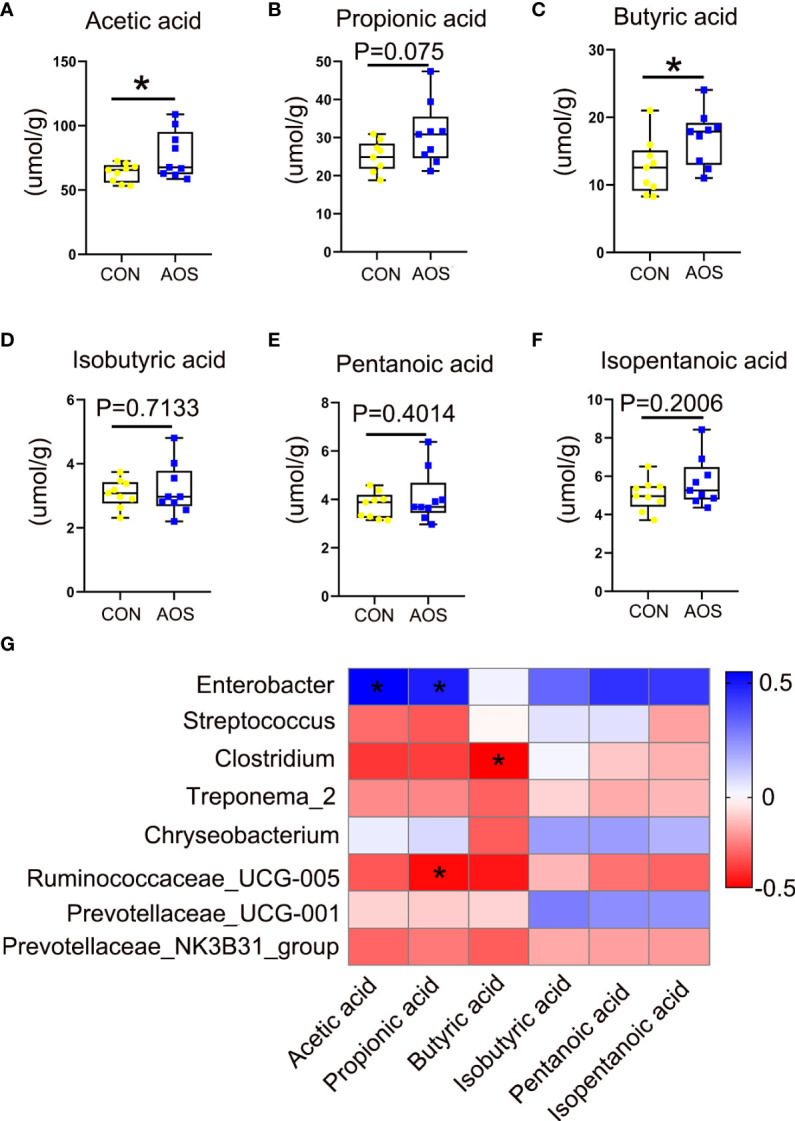
AOS improved the content of short-chain fatty acids in aging boar feces. **(A)** Acetic acid level in feces. **(B)** Propionic acid level in feces. **(C)** Butyric acid level in feces. **(D)** Isobutyric acid level in feces. **(E)** Pentanoic acid level in feces. **(F)** Isopentanoic acid level in feces. **(G)** The correlation between gut microbes and SCFAs. Data were expressed as the mean ± SEM. The *y*-axis represents the relative amount. The *x*-axis represents the treatments (*n* = 9/group). ^*^ *p* < 0.05.

To analyze the metabolites of gut microbes, we measured the SCFAs in the feces of aging boars. Some SCFAs were significantly increased after feeding AOS such as acetic acid (**Figure 5A**; *p* < 0.05) and butyric acid (**Figure 5C**; *p* < 0.05). The propionic acid has a tendency to increase (**Figure 5B**; *p =* 0.075). After feeding AOS, some short-chain fatty acids also increased, but the differences were not significant, such as for isobutyric acid (**Figure 5D**; *p =* 0.7133), pentanoic acid (**Figure 5E**; *p =* 0.4014), and isopentanoic acid (**Figure 5F**; *p =* 0.2006). There was a good correlation between microorganisms and short-chain fatty acids (**Figure 5G**). *Enterobacter* was significantly positively correlated with acetic acid and propionic acid, respectively. *Ruminococcaceae_UCG-005* was significantly negatively correlated with propionic acid. Meanwhile, *Clostridium* was significantly negatively correlated with butyric acid.

### Spearman correlation among fecal microbes, sperm metabolites, and sperm parameters

**Figure 6:**
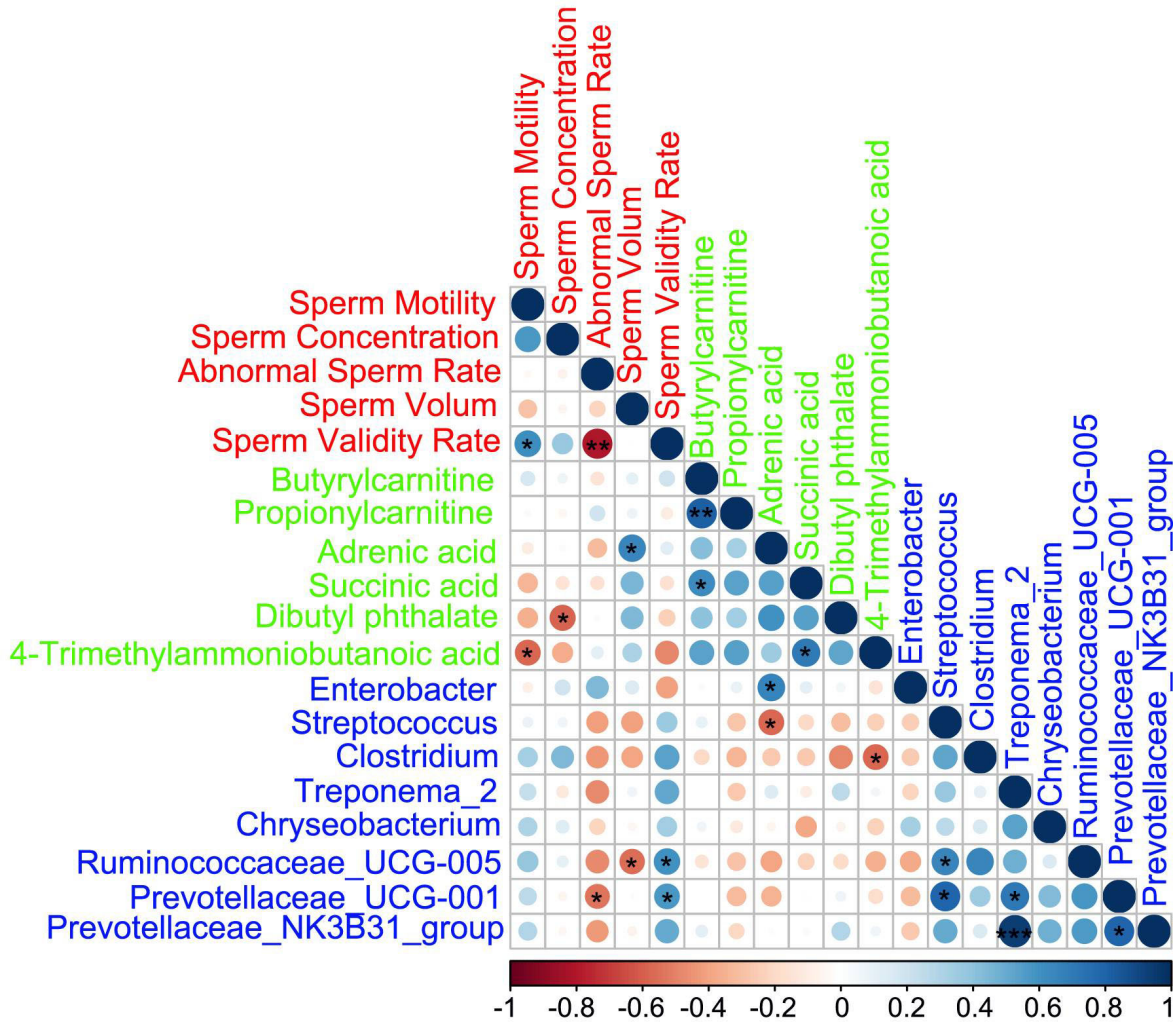
Correlations among fecal microbes, sperm metabolites, and sperm parameters. Red represents sperm parameters, green represents sperm metabolites, and blue represents gut microbes. Blue cycles represent positive correlation and red cycles represent negative correlation. The size of the circle represents the strength of the correlation (larger circle = stronger correlation). ^**^ *p* < 0.01, ^*^ *p* < 0.05.

The Spearman correlation analysis (**Figure 6**; **Table S6**) indicated that the fecal microbiota, sperm metabolites, and semen parameters were well correlated. First, the sperm metabolites and gut microbes were well correlated with each other. Second, there was a good correlation between sperm metabolites and gut microbes. In terms of semen quality, the decreased metabolite dibutyl phthalate in the AOS group was significantly negatively with sperm concentration. The *Ruminococcaceae_UCG-005* was positively correlated with sperm validity rate and negatively correlated with sperm volume. Meanwhile, *Prevotellaceae_UCG-001* was positively correlated with sperm validity rate and negatively correlated with abnormal sperm rate.

## Discussion

As a natural plant extract, AOS has multiple biological functions, the most prominent being antioxidant (Yang et al., 2022) and anti-aging (Feng et al., 2021). There is no doubt that according to the literature reports, AOS can improve the sperm motility of young boars (Han et al., 2022). In the study of mice as an experimental animal model, AOS can repair testicular damage caused by busulfan (Zhao et al., 2020c; Zhang et al., 2021b; Yan et al., 2022), thereby improving sperm quality. In this study, by feeding AOS for aging boars, sperm parameters had been significantly improved, and the testosterone content in the blood had also been significantly increased. As we all know, testosterone is an important hormone that can regulate spermatogenesis (Smith and Walker, 2014; Ge et al., 2021; Walker, 2021) and promote sexual desire (van Anders, 2012; Uloko et al., 2022); it has a positive effect on improving the lifespan of aging boars. At the same time, AOS can significantly improve the protein content in boar sperm, such as Catsper 8 and PKA. Studies have shown that Catsper 8 can maintain the chromatin integrity and morphology of sperm (Carlson et al., 2009; Khordad et al., 2022). However, PKA not only can participate in the fertilization process of mammals, especially sperm capacitation (Baro Graf et al., 2020), but also is highly associated with sperm motility and apoptosis (Huang et al., 2018; Yan et al., 2020). It can be seen that the potential mechanism of AOS extended the lifespan of the aging boar and improved the key protein content in the sperm and the blood testosterone level.

Sperm metabolites play a vital role in the procedure of spermatogenesis (Zhao et al., 2022). Studies have shown that AOS was conducive to improving the blood metabolome of boars, which, in turn, affects systemic metabolism (Han et al., 2022; Hao et al., 2022). At the same time, AOS was also conducive to improving metabolites in boar sperm. In the experiment involving young boars, AOS could improve some unsaturated fatty acids, amino acids, and antioxidants to improve semen quality (Han et al., 2022). In this study, AOS improved the sperm metabolome of aging boars; several types of fatty acids and derivatives were significantly increased, such as butyrylcarnitine, propionylcarnitine, and adrenic acid. A large number of studies indicated that the butyrylcarnitine content in the study of Bulls was significantly positively correlated with the reproductive performance (Longobardi et al., 2020). However, in the male reproductive tract, propionylcarnitine was secreted by the epididymis, which will protect sperm in semen (Golan et al., 1983). Through the study of the metabolome on mice testicles, the adrenic acid content was significantly positively correlated with testicular function (Lai et al., 2017). AOS could significantly increase the antioxidant content in boar semen such as succinic acid. Studies have shown that succinic acid was a strong antioxidant that improves sperm motility by reducing the content of ROS in epididymis (Frenkel et al., 1975; Nikolopoulou et al., 1985). In contrast, AOS could also reduce sperm metabolite dibutyl phthalate, which was a substance that was not conducive to spermatogenesis (Czubacka et al., 2021). A study has also shown that dibutyl phthalate induced oxidative stress and impaired spermatogenesis in adult rats (Aly et al., 2016). Therefore, the potential mechanism of AOS extending the lifespan of aging boars was to improve the metabolites in the sperm, thereby improving the semen quality.

As the largest digestive organ of animals, the intestine has become a research hotspot in recent years (Takiishi et al., 2017; Zhou et al., 2020). As an important medium between the meal and the host, intestinal microorganisms not only regulate the health of the host, but also generate a specific connection with the organs, such as the intestine–liver axis (De Gregorio et al., 2020) and the intestine–testicular axis (Zhao et al., 2020b). Therefore, it plays a vital role in human and animals. In the study of young boars as a model, it was found that AOS could improve the composition of intestinal flora. On the one hand, it improved the relative abundance of beneficial bacteria such as *Butyricicoccus* and *Bifidobacterium*; on the other hand, it reduced the relative abundance of harmful bacteria such as *Streptococcus* and *Oscillibacter* (Han et al., 2022). In a research study with mice as the animal model, the semen quality was improved by using fecal microbial transplantation (FMT) technology (Yan et al., 2022; Sheng et al., 2023). In this study, because the experimental animals were older boars, only one beneficial type of bacteria—*Enterobacter*—has been increased. A study has shown that *Enterobacter* could improve the storage time of boar semen at room temperature (Prieto-Martínez et al., 2014). At the same time, *Prevotella* in a research study on reproduction was recognized as a kind of harmful bacteria, which was negatively related to sperm quality (Farahani et al., 2021). In the study, we found that both *Prevotellaceae_UCG-001* and *Prevotellaceae_NK3B31_group* were significantly reduced. Studies have shown that both bacteria could promote intestinal inflammation and be harmful to intestinal health (Huang et al., 2021; Wu et al., 2023). Therefore, AOS extended the service lifespan of the older boars by reducing the relative abundance of harmful bacteria. Meanwhile, short-chain fatty acids have a strong effect in the intestine. It can provide energy for small intestinal epithelial cells, which will affect the permeability of the intestinal mucosa (Hu et al., 2018; He et al., 2020; Liu et al., 2021). Intestinal bacteria can use glucose to produce butyric acid, and the energy provided by butyric acid can be used for spermatogenesis (Du et al., 2013; Yan et al., 2022). In this study, by determining the short-chain fatty acids in boar feces, we found that the content of acetic acid and butyric acid was significantly increased. The relative abundance of the changed microbes and short-chain fatty acids was well correlated. Therefore, the potential mechanism of AOS will improve the semen quality of aging boars and the composition of microbes in the intestine, thereby increasing the SCFA content to promote spermatogenesis.

## Conclusion

Adding 10 mg/kg AOS to the diet can extend the service lifespan of aging breeding boars by improving the intestinal microorganisms and sperm metabolites in a Duroc model. Therefore, AOS can be used as a feed additive to solve the productive problem with a high elimination rate of boar studs.

## Data availability statement

The raw data of 16s RNA sequencing had been uploaded to the NCBI SRA database with the accession number PRJNA1044847.

## Ethics statement

The animal experiments were followed by the Animal Care and Use Committee of the Institute of Animal Sciences of CAAS (IAS2022-24). The studies were conducted in accordance with the local legislation and institutional requirements. Written informed consent was obtained from the owners for the participation of their animals in this study.

## Author contributions

YZ: Data curation, Investigation, Writing – original draft, Writing – review & editing. ZW: Data curation, Methodology, Software, Writing – original draft. JT: Investigation, Resources, Writing – original draft. HS: Investigation, Resources, Writing – original draft. HJ: Investigation, Resources, Writing – original draft. YG: Methodology, Software, Writing – review & editing. HZ: Funding acquisition, Methodology, Project administration, Resources, Writing – review & editing. MS: Methodology, Project administration, Writing – review & editing.

## Supplementary material

Supplementary Table 1 Composition and nutrient analysis of basal diet.

Supplementary Table 2 Primary antibody.

Supplementary Table 3 Sperm metabolites data file.

Supplementary Table 4 KEGG metabolic pathway enrichment data file.

Supplementary Table 5 Fecal microbiota (Top 30) on genus level data file.

Supplementary Table 6 Spearman correlation data file.
