# Supplementary material for: Alginate oligosaccharide extends the service lifespan by improving the sperm metabolome and gut microbiota in an aging Duroc boars model
Source: Front Cell Infect Microbiol. 2023 Dec 5;13:1308484. doi: 10.3389/fcimb.2023.1308484 (PMC10728478; doi:10.3389/fcimb.2023.1308484)
Supplement: Supplementary Table 2 — Primary antibody. [file Table_2.docx]

**Table S2.** Primary antibody information

| **Name** | **Cat. #** | **Source (Animal)** | **Company** |
| --- | --- | --- | --- |
| Bcl | bs-0520R | Rabbit (polyclonal) | Beijing Biosynthesis Biotechnology CO. |
| PKA | bs-1645R | Rabbit (polyclonal) | Beijing Biosynthesis Biotechnology CO. |
| BAX | Bs-0127R | Rabbit | Beijing Biosynthesis Biotechnology CO. |
| CATSPER 8 | bs-23326R | Rabbit | Beijing Biosynthesis Biotechnology CO. |
